# Maternal RSV vaccination: clinical efficacy, safety, and global policy considerations: a systematic review

**DOI:** 10.1080/20523211.2026.2728239

**Published:** 2026-09-17

**Authors:** Khawla Abu Hammour, Qusai Manaseer, Murad Dweik, Walid Abu Hammour, Mohammed Zawiah, Faris El-dahiyat, Rana K. Abu-Farha

**Affiliations:** a Department of Clinical Pharmacy and Biopharmaceutics, Faculty of Pharmacy, The University of Jordan, Amman, Jordan; b Orthopedic Department, School of Medicine, The University of Jordan, Amman, Jordan; c Clear Lake Specialties, Webster, TX, USA; d School of Medicine, The University of Jordan, Amman, Jordan; e College of Medicine, Mohammed Bin Rashid University of Medicine and Health Science, Dubai, United Arab Emirates; f Department of Pharmacy Practice, College of Clinical Pharmacy, Hodeidah University, Al Hodeidah, Yemen; g Clinical Pharmacy Program, Al Ain University, Al Ain, United Arab Emirates; h Department of Clinical Pharmacy, Faculty of Pharmacy, Applied Science Private University, Amman, Jordan

**Keywords:** Respiratory syncytial viruses, respiratory tract infections, infant, vaccination

## Abstract

**Background:**

Respiratory syncytial virus (RSV) is among the leading causes of lower respiratory tract illness in newborns worldwide. This study reviews evidence on the efficacy and safety of maternal RSV vaccination.

**Methods:**

PROSPERO registered this PRISMA-compliant systematic review (CRD42024566335). Keywords associated with RSV, respiratory infections, newborns, and immunisation were used in a search of PubMed/MEDLINE, Embase, Scopus, Web of Science, and Cochrane CENTRAL (inception to mid-2026). Because of the heterogeneity of the studies, a descriptive synthesis of the safety and efficacy data was performed instead of a formal meta-analysis.

**Results:**

Sixteen publications – four real-world studies and twelve generated from nine separate parent trials/datasets (including three subgroup or subset analyses and post-hoc studies from two RCTs) – met the inclusion criteria. Depending on the dose, formulation, and length of follow-up, maternal immunisation was linked to a lower incidence of severe RSV-related lower respiratory tract infections in newborns, with efficacy/effectiveness varying from roughly 39% to 100%. Trial-based efficacy results were generally in line with real-world effectiveness data from national and multicenter initiatives in the US, UK, and Argentina. Preterm birth outcomes differed depending on the formulation: a bivalent RSVpreF vaccine (Pfizer MATISSE trial; [Li, Y., Wang, X., Blau, D. M., Caballero, M. T., Feikin, D. R., Gill, C. J., Madhi, S. A., Omer, S. B., Simões, E. A. F., & Campbell, H. (2022). Global, regional, and national disease burden estimates of acute lower respiratory infections due to respiratory syncytial virus in children younger than 5 years in 2019: A systematic analysis. *The Lancet*, *399*(10340), 2047–2064; Otsuki, T., Akada, S., Anami, A., Kosaka, K., Munjal, I., Baber, J., Shoji, Y., Aizawa, M., Swanson, K. A., & Gurtman, A. (2024). Efficacy and safety of bivalent RSVpreF maternal vaccination to prevent RSV illness in Japanese infants: Subset analysis from the pivotal randomized phase 3 MATISSE trial. *Vaccine*, *42*(22), 126041]) did not exhibit this association in post-hoc analyses, while an unadjuvanted RSVpreF vaccine (GSK; [Dieussaert, I., Hyung Kim, J., Luik, S., Seidl, C., Pu, W., Stegmann, J.-U., Swamy, G. K., Webster, P., & Dormitzer, P. R. (2024). RSV prefusion F protein–based maternal vaccine – preterm birth and other outcomes. *New England Journal of Medicine*, *390*(11), 1009–1021]) showed a significant increase in preterm birth (RR 1.37, 95% CI 1.08–1.74). Although post-vaccination responses were frequent, they were usually mild to moderate.

**Conclusion:**

There was no discernible rise in major adverse outcomes for mothers or newborns. Real-world data from a variety of situations now supports the conclusion that maternal RSV vaccination consistently decreased newborn RSV hospitalisation and severe disease. However, ongoing post-marketing surveillance and formulation-specific safety reporting are necessary due to variations in preterm birth risk that rely on timing and formulation.

## 1. Introduction

Respiratory syncytial virus (RSV) is one of the most common causes of lower respiratory tract disease in infants globally (Modjarrad et al., 2015). Most children are infected with RSV at least once during the first two years of life (Glezen et al., 1981). This infection is responsible for nearly four million hospitalisations and more than 100,000 deaths in children aged 5 years or less annually, and severe illness is usually associated with comorbidities such as asthma later in life (Li et al., 2022; Nair et al., 2010; Wu & Hartert, 2011). Mortality rates are significantly increased in countries classified as low- and middle-income countries (LMICs) (Wu & Hartert, 2011).

Several studies have assessed knowledge and attitudes regarding RSV vaccination (Abu Hammour et al., 2025; Gavaruzzi et al., 2025; Hammour et al., 2026), but because there is insufficient time after birth for an infant's own immune system to mount protective immunity (Johnson et al., 1987; Olmsted et al., 1986; Stott et al., 1987), passive immunisation through maternal vaccination has been proposed as an alternative strategy to prevent severe RSV infection during early infancy (Li et al., 2022; Nair et al., 2010; Sheffield et al., 2013).

Passive immunisation, in which maternally derived IgG antibodies cross the placenta to protect the infant, has previously been used against other pathogens, including influenza and coronavirus (Abu-Raya et al., 2020; Aranda & Polack, 2019; Halasa et al., 2022). In order to prevent serious RSV disease in their unborn children, the World Health Organization advises pregnant women to receive the respiratory syncytial virus (RSV) vaccine (WHO, 2024).

Immunogenicity and safety data for several maternal RSV vaccine candidates have accumulated in recent years; however, published randomised controlled trials (RCTs) vary considerably in design, formulation, and outcome reporting, limiting direct comparison. Evidence has been summarised in two significant secondary syntheses, a Cochrane systematic review of RCTs (Phijffer et al., 2024) and a rapid meta-analysis focused on preterm birth (Kuitunen et al., 2025). However, neither of these summaries took into account the increasing amount of post-licensure, real-world effectiveness and safety data that are currently available from national immunisation programs outside of the United States. Therefore, it is necessary to comprehensively assess the safety and effectiveness of RSV vaccination during pregnancy, incorporating real-world observational data and clinical trial data from a variety of nations and healthcare environments. The aim of the present study is to systematically review the evidence regarding both the efficacy and the safety of maternal RSV vaccination.

## 2. Method

### 2.1. Study registration

This systematic review was registered in the PROSPERO database (CRD42024566335), and analysis was reported in adherence to the Preferred Reporting Items for Systematic Reviews (PRISMA) statement guidelines (Page et al., 2021).

### 2.2. Search strategy

Five electronic databases: PubMed/MEDLINE, Embase, Scopus, Web of Science, and Cochrane CENTRAL were searched from database inception to mid- 2026. Controlled vocabulary (MeSH/Emtree) and free-text terms relating to respiratory syncytial virus, vaccination, and maternal immunisation were combined. Only human studies and English-language publications were included in the searches. To improve coverage and lessen publication bias, the reference lists of the included publications were manually searched, and an additional Google Scholar search was carried out.

### 2.3. Eligibility criteria and study selection

**Figure 1.**
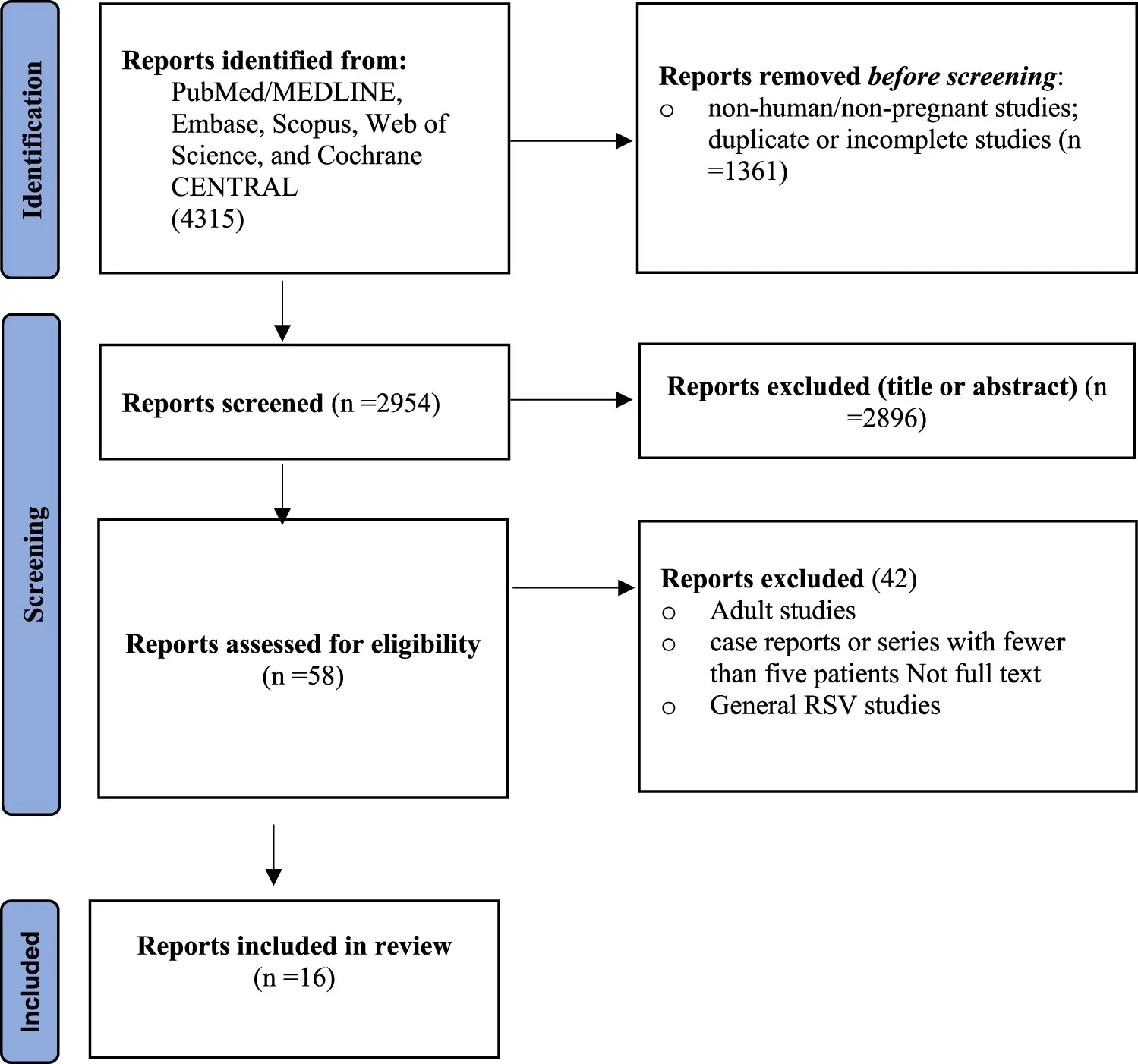
*PRISMA* flow chart of the systematic review.

Studies that reported at least one predetermined effectiveness or safety outcome in mothers or infants, were published in English, and included pregnant women vaccinated against RSV and their babies were eligible. Research involving non-human subjects, non-pregnant women, adults not involved in maternal or infant RSV immunisation, case reports or case series with fewer than five patients, editorials, letters, or conference abstracts without extractable full data, general RSV epidemiology or vaccine studies that did not specifically address maternal immunisation, duplicate publications, or ongoing or incomplete studies were all excluded. The exclusion grounds displayed in the PRISMA flow diagram (Figure 1) are directly correlated with these categories.

### 2.4. Data extraction and outcomes

Two authors (A.K., A.R.) independently screened studies according to the predetermined criteria: abstracts were screened first, followed by full-text assessment of eligible records. Risk of bias was assessed independently by two reviewers, and data were extracted independently by the same two authors; discrepancies were resolved via discussion or adjudication by a third author (A.W.).

Neonatal outcomes assessed included RSV-associated severe lower respiratory tract illness (LRTI), hospitalisation, and mortality; maternal outcomes assessed included preterm birth, preeclampsia, stillbirth, and reactogenicity. Participants were not double-counted in aggregate totals when two or more publications (such as a primary report and a later post-hoc or subgroup analysis) reported on the same parent trial. This link is expressly indicated in Table 1. To support the duration-of-protection study, the gestational-age window in which vaccination was given (such as the currently advised 32–36 weeks) and the follow-up period for infant outcomes (such as 90 versus 180 days) were also extracted where reported. A formal meta-analysis and formal assessment of publication bias were not undertaken given the small number of included studies and the heterogeneity of study designs and vaccine formulations; therefore, rather than using pooled effect estimates, findings are summarised descriptively.

**Table 1.** Characteristics of the included studies (sixteen publications; rows added following peer review are marked in yellow).

Study (Author, Year)	Study design	Phase	Study period	Location	Sponsor
Muňoz et al. (2003)	RCT	N/A	N/A	USA	Wyeth Lederle
Muñoz et al. (2019)	RCT	Phase 2	2014–2015	USA	Novavax
Madhi et al. (2020) – parent trial^a^	RCT	Phase 3	2015–2018	11 countries	Novavax
Lewnard et al. (2022) – post-hoc of Madhi et al. (2020)^a^	Post-hoc secondary analysis	Phase 3	2015–2018	11 countries	Bill & Melinda Gates Foundation
Simões et al. (2022)	RCT	Phase 2b	2019–2020	USA, Chile, Argentina, South Africa	Pfizer
Bebia et al. (2023)	RCT	Phase 2	2019–2021	9 countries	GSK
Kampmann et al. (2023) – MATISSE parent trial^b^	RCT	Phase 3	2020–2022	18 countries	Pfizer
Li et al. (2022) – post-hoc of Kampmann et al. (2023)^b^	Post-hoc analysis (preterm birth)	Phase 3	2020–2022	18 countries	Pfizer
Otsuki et al. (2024) – Japan subset of Kampmann et al. (2023)^b^	Subset analysis	Phase 3	2020–2022	Japan	Pfizer
Dieussaert et al. (2024)	RCT	Phase 3	2020–2022	24 countries	GSK
McLachlan et al. (2025)	Case-control (observational)	–	2024–2025	Scotland, UK	None reported
Williams et al. (2025)	Case-control (observational)	–	2024–2025	UK	Respiratory Syncytial Virus Consortium in Europe
Son et al. (2024)	Observational cohort	–	2023–2024	USA (New York City, 2 centres)	Non-commercial / institutional
Pérez Marc et al. (2025)	Multicentre, retrospective, test-negative case-control	–	2024 (national programme)	Argentina (12 hospitals)	Non-commercial
Moline et al. (2026)	Population-based surveillance, test-negative case-control	–	Oct 2024–Apr 2025	USA (7 pediatric centres)	CDC / non-commercial
Gabet et al. (2025)	Nationwide matched cohort (claims database)	–	Sep–Dec 2024 (2024–2025 campaign)	France (national)	Non-commercial (EPI-PHARE)

^a^ Same parent RCT: Lewnard et al. (2022) is a post-hoc secondary analysis of the trial reported by Madhi et al. (2020) (Novavax adjuvanted RSV F protein vaccine); participants are the same and are not double-counted. ^b^Same parent RCT (MATISSE): Madhi et al. (2025) and Otsuki et al. (2024) are post-hoc/subset analyses of the trial reported by Kampmann et al. (2023) (Pfizer bivalent RSVpreF vaccine); participants are not double-counted.

## 3. Results

The literature search conducted through mid- 2026 retrieved 4,315 records. After title/abstract and full-text screening, sixteen publications met the original eligibility criteria (Bebia et al., 2023; Dieussaert et al., 2024; Gabet et al., 2025; Kampmann et al., 2023; Lewnard et al., 2022; Li et al., 2022; Madhi et al., 2020; McLachlan et al., 2025; Moline et al., 2026; Muňoz et al., 2003, 2019; Otsuki et al., 2024; Pérez Marc et al., 2025; Simões et al., 2022; Son et al., 2024; Williams et al., 2025), bringing the total to sixteen included publications. The search and selection process is shown in the PRISMA flow diagram (Figure 1), and study characteristics are summarised in Table 1.

### 3.1. Characteristics of included studies

Sixteen publications spanning a 23-year period (2003–2026) were included. These include three post-hoc, subgroup, or subset analyses, two independent observational case–control studies, and seven original study reports (six RCTs plus the Madhi et al. (2020) trial, which is also the parent study for a derivative analysis) (McLachlan et al., 2025; Williams et al., 2025). Four real-world studies were conducted: a nationwide French claims-database safety study of vaccinated women matched to unvaccinated controls (Gabet et al., 2025); a 12-hospital, national, test-negative case–control effectiveness study from Argentina covering the first season of a national maternal immunisation program (Pérez Marc et al., 2025); a 7-center US population-based surveillance study of vaccine effectiveness and population impact (Moline et al., 2026).

A parent trial is shared by two families of publications. First, the post-hoc secondary analysis of antimicrobial prescribing published by Lewnard et al. (2022) is based on the Novavax adjuvanted RSV F protein vaccination trial reported by Madhi et al. (2020); these two publications describe the same participants and are counted as one trial. Second, the post-hoc preterm-birth analysis by Li et al. (2022) and the Japanese subset analysis by Otsuki et al. (2024) are derived from the MATISSE trial (Pfizer bivalent RSVpreF vaccine) reported by Kampmann et al. (2023); the participants in these two derivative publications are a subset of the totals reported by Kampmann et al. (2023).

Thirteen independent trials/datasets are represented by the sixteen publications, counting each parent trial once: Muñoz et al. (2003, 2019); the Madhi et al. (2020)/Lewnard et al. (2022) trial; Simões et al. (2022); Bebia et al. (2023); the Kampmann et al. (2023)/Li et al. (2022)/Otsuki et al. (2024) MATISSE trial; Dieussaert et al. (2024); McLachlan et al. (2025); Williams et al. (2025); Son et al. (2024); Pérez Marc et al. (2025); Moline et al. (2026); and Gabet et al. (2025).

An estimated 18,000 mother-infant pairs were enrolled in the seven RCTs across the nine trials/datasets, and an additional 31,000 mother-infant pairs contributed data through the two original observational case–control studies (UK and Scotland), for a total of about 49,000 participants. Four further research studies make significant additional contributions: 5,029 children under two years old from seven US centers (Moline et al., 2026); 2,973 pregnant people (Son et al., 2024); and 29,032 vaccinated women matched to 24,891 unvaccinated women (Gabet et al., 2025). When all are combined, the review now has a total of well over 110,000 participants.

Studies were conducted in single countries and in large multinational trials spanning up to 24 countries. Early trials were based in the United States, while more recent trials were distributed globally. In addition to the two original observational case–control studies that provided real-world effectiveness data from the UK and Scotland, a nationwide claims-database analysis of the 2024–2025 immunisation campaign in France provided the largest maternal safety dataset to date (Gabet et al., 2025). Argentina was the first country to implement a national maternal RSVpreF immunisation program as primary RSV-prevention policy, starting in March 2024 (Pérez Marc et al., 2025). Population-level effects on RSV hospitalisation rates during the 2024–2025 season were also found by another US multicenter surveillance research study (Moline et al., 2026).

Most RCTs included in the current review were industry-sponsored (Pfizer, GSK, Novavax); the included observational studies reported non-commercial funding.

### 3.2. Design of the included studies, interventions, and included population

Included studies assessed maternal RSV vaccination administered primarily during the late second or third trimester to women with singleton, healthy pregnancies. Most RCTs compared RSV prefusion F (RSVpreF) protein vaccines, at doses ranging from 60 µg to 240 µg, against placebo.

Early-phase studies enrolled small samples (20–170 mother-infant pairs) to assess safety and immunogenicity, while later trials enrolled more than 7,000 pregnant women and infants. Sample sizes were generally comparable between intervention and placebo arms within each trial.

From birth, infants in the big multicenter studies were monitored for outcomes related to RSV. Real-world data was provided by the two original observational case–control studies: a national study in Scotland including over 27,000 vaccinated pregnant women and a study in the UK involving mother-infant pairings that were both RSV-positive and RSV-negative. Studies go beyond these to broaden the body of evidence: a 12-hospital BERNI test-negative case–control effectiveness study from Argentina's first national maternal immunisation season (Pérez Marc et al., 2025); a 7-center US population-based surveillance study of vaccine effectiveness and population impact (Moline et al., 2026); a national French claims-database safety study of over 29,000 vaccinated women (Gabet et al., 2025). Full details are provided in Table 2.

**Table 2.** Overview of study design, interventions, and participants in included studies.

Study (authors) Year of publication	Arms	Sample size		Study participants
		Mothers	Infants	
Muňoz et al. (2003)	Adjuvanted RSV F protein (50 µg)	20	20	Singleton healthy pregnant women (third trimester)
	placebo	15	15	
Muňoz et al. (2019)	Adjuvanted RSV F protein (120 μg)	22	22	Singleton healthy pregnant women (third trimester)
	Placebo	28	28	
Madhi et al. (2020)	Adjuvanted RSV F protein (120 μg)	3,051	3,029	Singleton healthy pregnant women (third trimester)
	Placebo	1558	1574	Formulation buffer (without aluminum)
Simões et al. (2022)	Unadjuvanted RSVpreF (120 μg)	79	79	Singleton healthy pregnant women (late second and third trimesters)
	Aluminum hydroxide adjuvant only (no RSVpreF antigen)	84	84	
	Unadjuvanted RSVpreF (240 μg)	78	77	
	Adjuvanted RSVpreF (240 μg)	86	85	
	Placebo	79	78	
Bebia et al. (2023)	unadjuvanted RSVpreF (60 μg)	70	67	Singleton healthy pregnant women (third trimester)
	unadjuvanted RSVpreF (120 μg)	75	73	
	Placebo	68	66	
Kampmann et al. (2023)	unadjuvanted RSVpreF (120 μg)	3695	3570	Singleton healthy pregnant women (late second and third trimester)
	Placebo	3697	3558	
Dieussaert et al. (2024)	unadjuvanted RSVpreF (120 μg)	3557	3494	Singleton healthy pregnant women (late second and third trimester)
	Placebo	1771	1739	
Lewnard et al. (2022)	Adjuvanted RSV F protein (120 μg)	3,051	3,029	Singleton healthy pregnant women (third trimester)
	Placebo	1558	1574	
Otsuki et al. (2024)	unadjuvanted RSVpreF (120 μg)	230	218	
	placebo	232	216	
Li et al. (2022)	unadjuvanted RSVpreF (120 μg)	3695	3570	Singleton healthy pregnant women (late second and third trimester)
	Placebo	3697	3558	
McLachlan et al. (2025)	Vaccinated	27,565 pregnant 12747 vaccinated more than 14 d before delivery	354 infants aged 90 days or younger had RSV- related LRTI hospital admissions	
	Control	3511	1518	
Williams et al. (2025)	RSVpreF (120 μg)	391 mother-pair RSV- positive cases		
146 mother – infant pair negative controls				
Son et al. (2024)	RSVpreF (unadjuvanted bivalent) vs unvaccinated	1,026 vaccinated / 1,947 unvaccinated (2,973 total)	–	Singleton pregnancies, during the 2023–2024 recommended vaccination period, were vaccinated mostly at ∼34 weeks’ gestation
Pérez Marc et al. (2025)	RSVpreF (national programme) vs unvaccinated	–	Infants ≤6 months hospitalised with LRTD (test-negative design, 12 hospitals)	Vaccination per Argentine national authorisation, 32 + 0/7–36 + 0/7 weeks’ gestation
Moline et al. (2026)	Maternal RSVpreF vs unvaccinated; nirsevimab vs no product	–	5,029 children <2 years with medically attended ARI, 7 US centres	Newborns/infants <6 months (maternal vaccine exposure) and <8 months (nirsevimab exposure)
Gabet et al. (2025)	RSVpreF vs unvaccinated (1:1 matched)	29,032 vaccinated; 24,891 matched pairs	–	Singleton and multiple pregnancies ≥22 weeks’ gestation, France, 2024–2025 campaign, recommended a 32–36-week window

### 3.3. Outcomes

Across the included studies, maternal RSV vaccination was associated with a decrease in RSV-related hospitalisation and severe LRTI in newborns, with vaccination efficacy or effectiveness estimates varying from roughly 39% to 100% based on vaccine formulation, dose, and follow-up time. The results below are presented by research; no formal pooled effect estimate was computed.

### 3.4. Neonatal efficacy and safety

RSV-associated severe LRTI was assessed in eleven publications (Bebia et al., 2023; Dieussaert et al., 2024; Kampmann et al., 2023; Li et al., 2022; Madhi et al., 2020; McLachlan et al., 2025; Muňoz et al., 2003, 2019; Otsuki et al., 2024; Simões et al., 2022; Williams et al., 2025). Estimates of vaccine efficacy and efficiency against severe or medically attended RSV-LRTI included the following: 57.1% (99.5% CI 14.7–79.8) for medically attended RSV-associated LRTI within 90 days; 81.8% (99.5% CI 40.6–96.3) at 90 days and 69.4% (97.58% CI 44.3–84.1) at 180 days for medically attended severe LRTI at the follow-up reported by Madhi et al. (2020).

In Kampmann et al. (2023), 69.0% (95% CI 33.0–87.6) for severe RSV-LRTI; in Dieussaert et al. (2024), 100% (95% CI 30.9–100.0) at 90 days and 87.6% (95% CI 7.2–99.7) at 180 days for medically attended RSV-LRTI in the small Japanese MATISSE subset (Otsuki et al., 2024); and 81.0% (95% CI 68.6–88.5) against hospital admission in the Scottish cohort (McLachlan et al., 2025), and in the UK case–control study, 72% (95% CI 48–85) against hospitalisation (Williams et al., 2025).

Five original-search papers with about 13,000 participants in our sample specifically evaluated hospitalisation related to RSV as an outcome (Bebia et al., 2023; Kampmann et al., 2023; Madhi et al., 2020; Simões et al., 2022; Williams et al., 2025). The resulting effect estimates, which range from 44.4% to 81.0% depending on research and follow-up period, show a consistent decrease in RSV-associated hospitalisation across formulations. These conclusions are supported by real-world efficacy data from four additional investigations. The 7-center US surveillance study estimated maternal vaccine effectiveness of 70% against RSV-associated hospitalisation (compared with 81% for nirsevimab over the same period), with population-level RSV hospitalisation rates in infants younger than 12 months falling by up to half compared with pre-introduction seasons (Moline et al., 2026).

The BERNI study reported high effectiveness against RSV-associated LRTD and severe LRTD leading to hospitalisation, sustained from birth to 6 months of age (Pérez Marc et al., 2025). Instead of directly adding to the LRTI efficacy count above, two additional original-search papers address related but different outcomes: Blauvelt et al. (2025), which reports vaccination and nirsevimab uptake rates rather than a comparable efficacy or safety outcome, and Lewnard et al. (2022), which reports on antibiotic prescribing.

Three original-search investigations with roughly 17,000 participants evaluated infant mortality (Dieussaert et al., 2024; Kampmann et al., 2023; Madhi et al., 2020). Dieussaert et al. (2024) were the only ones to report a specific effect estimate: neonatal death occurred in 0.4% (13/3,494) of infants in the vaccine group versus 0.2% (3/1,739) in the placebo group (relative risk 2.16, 95% CI 0.62–7.56, *p* = 0.23). This difference was not statistically significant, with a wide confidence interval indicating limited statistical power for this uncommon outcome. The other two trials did not consistently report mortality effect estimates. One study (Lewnard et al., 2022) examined the effect of maternal vaccination on antimicrobial prescriptions related to acute LRTI during early infancy and found a 12.9% decrease in antimicrobial prescriptions during the first three months of life; the source publication did not provide a confidence interval for this estimate. This result should not be extrapolated to other RSV vaccinations or considered an established primary outcome of maternal RSV immunisation because it is based on a single post-hoc examination of a particular vaccine formulation.

Overall, reported neonatal adverse events were predominantly mild to moderate and occurred at similar rates between vaccine and placebo groups; no consistent safety signal was identified for infants.

### 3.5. Maternal safety outcomes

Six studies involving roughly 17,500 pregnant women evaluated preterm birth (Bebia et al., 2023; Dieussaert et al., 2024; Kampmann et al., 2023; Madhi et al., 2020; Muňoz et al., 2019; Simões et al., 2022). Two large real-world safety studies were conducted: a nationwide French claims-database study of 29,032 vaccinated women matched to 24,891 unvaccinated women (Son et al., 2024). Results varied depending on the experiment and vaccination formulation.

Preterm birth was considerably more common in the vaccine group in the GSK unadjuvanted RSVpreF trial (relative risk 1.37, 95% CI 1.08–1.74, *p* = 0.01; Dieussaert et al., 2024). This finding led to the early termination of the vaccine candidate's clinical development. On the other hand, there was no statistically significant difference in preterm birth (relative risk 1.20, 95% CI 0.98–1.46; Li et al., 2022) according to the post-hoc analysis of the Pfizer bivalent RSVpreF (MATISSE) trial; the confidence interval crosses 1.0 and this cannot be considered a significant reduction.

Although this subset was not powered to detect differences in this outcome, a smaller Japanese MATISSE subset found numerically decreased preterm-birth rates in the vaccine arm (3.2% vs. 6.0%; Otsuki et al., 2024). A statistically significant difference between groups was not found in any other original-search study evaluating preterm birth.

Within the currently advised immunisation timeframe, two more studies offer far greater real-world assurance: Gabet et al. (2025) found no significant increase in preterm birth among women vaccinated at or before 32 weeks’ gestation, i.e. within the currently recommended window (weighted incidence rate ratio 1.13, 95% CI 0.98–1.31). Son et al. (2024) found no increased risk of preterm birth with nonadjuvanted bivalent RSVpreF (5.9% of vaccinated vs. 6.7% of unvaccinated participants).

Results of the other three studies, comprising approximately 10,000 participants, assessed the interval between vaccination and delivery (Bebia et al., 2023; Dieussaert et al., 2024; Madhi et al., 2020), revealed that no significant difference was observed between vaccinated and placebo groups.

Seven studies (∼18,000 participants) evaluated preeclampsia (Bebia et al., 2023; Dieussaert et al., 2024; Kampmann et al., 2023; Li et al., 2022; Madhi et al., 2020; Muňoz et al., 2019; Simões et al., 2022), and five studies evaluated stillbirth. No significant differences were found for either outcome. In the Gabet et al. (2025) cohort, stillbirth and hypertensive disorders of pregnancy were also evaluated as secondary outcomes; no discernible excess was seen.

No significant differences were observed for congenital abnormalities or intrauterine growth restriction across the studies assessing these outcomes.

Local and systemic reactogenicity was commonly reported and was generally mild to moderate. Local edema was more frequent in vaccinated participants in five studies (Bebia et al., 2023; Kampmann et al., 2023; Madhi et al., 2020; Muňoz et al., 2019; Simões et al., 2022); for example, local injection-site reactions occurred in 40.7% of vaccinated participants versus 9.9% of placebo recipients in Madhi et al. (2020). Local pain was more common in vaccinated participants in six studies (Bebia et al., 2023; Dieussaert et al., 2024; Kampmann et al., 2023; Madhi et al., 2020; Muňoz et al., 2003, 2019; Simões et al., 2022), and local erythema was more common in five studies (Bebia et al., 2023; Kampmann et al., 2023; Madhi et al., 2020; Muňoz et al., 2019; Simões et al., 2022).

Diarrhea, assessed in six studies, did not differ significantly between groups, whereas vomiting was reported more frequently among vaccinated participants. Joint and muscle pain (five studies) and headache (five studies) did not differ significantly between groups. Fatigue (six studies) was numerically more frequent following vaccination but did not reach statistical significance. Fever, assessed in six studies, did not differ significantly between groups (Table 3).

**Table 3.** Efficacy and safety outcomes of RSV vaccines.

Study (authors) Year of publication	Intervention			Conclusion	
	type	Dose	Time of administration	Efficacy	Safety
Muňoz et al. (2003)	Purified F protein with aluminum hydroxide	Single dose (IM)	30–34 wk of gestation.	Infants born to vaccine recipients had higher concentrations of IgG Ab and anti-F IgG binding antibody to RSV at birth, 2 and 6 months of life (*P* < 0.01).	RSV vaccine was safe and well tolerated by pregnant women. Mild pain at the site of injection occurred more in vaccinated mothers than placebo recipients (*P* = 0.005). There were no systemic reactions, fever, or serious AEs associated with vaccine administration in mothers. There were no differences among the infants in both groups in perinatal or neonatal outcomes. There was no increase in the morbidity associated with respiratory tract illnesses in infants of vaccinated mothers.
	Saline				
Muňoz et al. (2019)	Purified F protein with aluminum phosphate	Single dose (IM)	33–35 wk of gestation.	Maternal immunisation was effective in protecting infants from RSV disease.	The vaccine was well tolerated; no meaningful differences in pregnancy or infant outcomes were observed between study groups. There was no evidence of severe RSV disease in infants of vaccinated mothers.
	Saline				
Madhi et al. (2020)	Purified F protein with aluminum phosphate	Single dose (IM)	28–36 wk of gestation.	Medically significant lower respiratory tract infection was 1 lower in the vaccine group compared to placebo group (vaccine efficacy, 39.4%; 97.52% CI, −1.0–63.7; 95% CI, 5.3–61.2). & for RSV-associated LRTIs with severe hypoxemia were 0.5% and 1.0% (vaccine efficacy, 48.3%; 95% CI, −8.2–75.3), and hospitalisation for RSV-associated LRTIs were 2.1% and 3.7% (vaccine efficacy, 44.4%; 95% CI, 19.6–61.5).	Local injection-site reactions were more common with vaccine than with placebo (40.7% vs. 9.9%), but other adverse events were similar in the two groups.
	Formulation buffer without aluminum				
Simões et al. (2022)	Bivalent purified prefusion F protein with/without aluminum hydroxide	Single dose (IM)	24–36 wk of gestation.	Infants of vaccinated mothers had similar titers in umbilical-cord blood and similar transplacental transfer ratios across the range of assessed gestational ages.	Most reactions were mild to moderate; The incidences of adverse events in the women and infants were similar in the vaccine and placebo groups.
Bebia et al. (2023)	Purified prefusion F protein	Single dose (IM)	28–33 wk of gestation.	Induced robust RSV-specific immune responses with successful antibody transfer to their newborns.	RSV vaccine was well tolerated. No pregnancy or neonatal AEs were considered vaccine/placebo-related.
	Saline				
Kampmann et al. (2023)	Bivalent purified prefusion F protein	Single dose (IM)	24–36 wk of gestation.	The incidence of medically attended severe LRTIs was higher within 90 days after birth in infants of the vaccinated mother group than in the placebo group (vaccine efficacy, 81.8%; 99.5% CI, 40.6 96.3). And 180 days (vaccine efficacy, 69.4%; 97.58% CI, 44.3–84.1). The incidence of medically attended RSV-associated LRT illness was lower within 90 days after birth in infants of women in the vaccine group than infants of women in the placebo group (vaccine efficacy, 57.1%; 99.5% CI, 14.7–79.8).	No safety signals were detected in maternal participants or in infants up to 24 months of age. The incidences of AEs reported within 1 month after injection or within 1 month after birth were similar in both groups (the vaccine group) (13.8% of women and 37.1% of infants) and the placebo group (13.1% and 34.5%, respectively).
	NA				
Dieussaert et al. (2024)	Purified prefusion F protein	Single dose (IM)	24–34 wk of gestation.	The incidence of any medically assessed RSV-associated LRT disease was lower in infants of vaccinated mothers (vaccine efficacy, 65.5%; 95% credible interval, 37.5–82.0), and also severe medically assessed RSV-associated LRT disease (vaccine efficacy, 69.0%; 95% credible interval, 33.0–87.6).	Preterm birth incidence was higher in the infants of the vaccinated group compared to placebo group (relative risk, 1.37; 95% confidence interval [CI], 1.08–1.74; *P* = 0.01); neonatal death occurred in 0.4% (13 of 3494) and 0.2% (3 of 1739), respectively (relative risk, 2.16; 95% CI, 0.62–7.56; *P* = 0.23). No other safety signal was observed.
	Sucrose reconstituted in NaCl solution				
Lewnard et al. (2022)	Purified F protein with aluminum phosphate	Single dose (IM)	28–36 wk of gestation.	Administration of RSV vaccine was associated with a reduction in antimicrobial prescribing among their infants by 12.9% over the first 3 months of life.	NA
	Formulation buffer (without aluminum)				
Otsuki et al. (2024)	Bivalent purified prefusion F protein	Single dose (IM)	24–34 wk of gestation.	Observed vaccine efficacy (95% CIs) against infant RSV-MA-LRTI within 90 and 180 days after birth was 100.0% (30.9, 100.0; RSVpreF, 0 cases; placebo, 7 cases) and 87.6% (7.2, 99.7; RSVpreF, 1 case; placebo, 8 cases), respectively. Vaccine efficacy (95% CIs) against severe RSV-MA-LRTI within 90 and 180 days was 100.0% (−140.9, 100.0; RSVpreF, 0 cases; placebo, 3 cases) and 75.1% (−151.5, 99.5; RSVpreF, 1 case; placebo, 4 cases), respectively.	No safety concerns were identified. AE rates ≤1 month after vaccination/birth were similar in the RSVpreF (maternal, 16.1%; infant, 48.6%) and placebo (19.8%; 50.5%) groups. Preterm birth rates were also similar (RSVpreF, 3.2%; placebo, 6.0%).
	NA				
Li et al. (2022)	Bivalent purified prefusion F protein	Single dose (IM)	28–34 wk of gestation (for cases).	Not applicable (safety-focused post-hoc analysis).	No statistically significant increase in preterm birth (RR 1.20, 95% CI 0.98–1.46 – CI crosses 1.0, non-significant); no clinically significant increase in low birth weight or neonatal hospitalisation.
	NA		28–33 wks for control		
McLachlan et al. (2025)	RESpreF	Single dose (IM)	Received RSVpreF more than 14 days before delivery.	Adjusted vaccine effectiveness was higher among infants born preterm (<37 weeks’ gestation; 89·9% [55·3–97·7]; *p* = 0·0025), also for term-born infants (≥37 weeks’ gestation; 81·5% [73·9–87·0]; *p* < 0·0001). Vaccine effectiveness against RSV-associated LRTI hospital admission was 81·0% (68·6–88·5; *p* < 0·0001).	NA
Williams et al. (2025)	RSVpreF	Single dose (IM)	Received RSVpreF more than 14 days before delivery.	The effectiveness of RSVpreF vaccination for preventing infant hospitalisation was 72% (48–85) for infants whose mothers were vaccinated more than 14 days before delivery	NA
Son et al. (2024)	Nonadjuvanted bivalent RSVpreF	Single dose (IM)	At 32–36 weeks’ gestation.	Not assessed (safety-focused cohort).	No increased risk of preterm birth with vaccination (5.9% vaccinated vs 6.7% unvaccinated); no significant differences in hypertensive disorders of pregnancy, stillbirth, SGA birth weight, or neonatal outcomes assessed.
Pérez Marc et al. (2025)	RSVpreF, national programme	Single dose (IM)	Between 32 and 36 weeks of gestation and 14 days or more before delivery.	High real-world vaccine effectiveness against RSV-associated LRTD and severe LRTD leading to hospitalisation, sustained from birth to 6 months of age.	Not a primary focus of this effectiveness study.
Moline et al. (2026)	Maternal RSVpreF; nirsevimab (comparator)	–	–	Maternal vaccine effectiveness 70% against RSV-associated hospitalisation (nirsevimab 81%); RSV-associated hospitalisation rates in infants 0–11 months fell by up to ∼50% vs pre-introduction seasons.	Not a primary focus of this effectiveness/impact study.
Gabet et al. (2025)	RSVpreF, national campaign	Single dose (IM)	Between 33 and 36 weeks’ gestation.	Not an efficacy study.	No significant increase in preterm birth among women vaccinated at or before 32 weeks (weighted IRR 1.13, 95% CI 0.98–1.31); no major maternal or neonatal safety signal identified across preterm birth, stillbirth, SGA birth weight, cesarean delivery, hemorrhage, and preeclampsia.

### 3.6. Duration of protection

In the two trials that reported both time points, efficacy against medically attended severe RSV-associated LRTI decreased between 90 and 180 days after birth: 81.8% (99.5% CI, 40.6–96.3) at 90 days versus 69.4% (97.58% CI, 44.3–84.1) at 180 days in Kampmann et al. (2023) in the Japanese MATISSE subset (Otsuki et al., 2024). The pooled MATISSE data showed a similar pattern for medically attended (any-severity) RSV-associated LRTI, with efficacy of 57.1% at 90 days declining to 51.3% at 180 days. This pattern is in line with the transplacentally acquired maternal IgG antibody gradually declining during the first few months of the infant's life.

A protective window centered in the first three to six months of life is generally consistent with real-world effectiveness evidence. From birth to three months of age, RSVpreF's effectiveness against RSV-associated hospitalisation was high in the BERNI study, and it continued to be significant – albeit somewhat diminished – through six months (Pérez Marc et al., 2025). When vaccination took place more than 14 days prior to delivery, the adjusted vaccine effectiveness against hospitalisation for infants aged ≤90 days in Scotland was 81.0% (95% CI 68.6–88.5) (McLachlan et al., 2025). In the UK case–control study, the effectiveness against hospitalisation under the same exposure definition was 72% (95% CI 48–85) (Williams et al., 2025).

The maternal vaccine effectiveness of 70% against RSV-associated hospitalisation during the October 2024–April 2025 season was estimated by the US multicenter surveillance study. This is somewhat lower than the 81% effectiveness estimated for nirsevimab during the same period. This is consistent with the assumed average duration of protection following maternal vaccination (on the order of 3–6 months) being generally shorter than that modelled for extended half-life monoclonal antibodies like nirsevimab (∼8–9 months) (Moline et al., 2026).

When combined, these findings show that maternal RSV vaccination provides robust but time-limited protection, concentrated in the first three to six months of life, and that the time between vaccination and delivery, as well as the timing of vaccination in relation to the start of the local RSV season, are significant factors in determining the level of protection an individual infant will receive. The best time to vaccinate during pregnancy is directly impacted by these factors.

## 4. Discussion

Although no formal meta-analysis or pooled effect estimate was carried out, this systematic review discovered that maternal RSV immunisation was consistently linked to less severe RSV-associated LRTI and decreased hospitalisation in newborns across various trial designs and vaccine formulations.

Across real-world observational studies and Phase 3 RCTs, maternal immunisation provided protection against severe RSV disease during early infancy, a period of high susceptibility and limited treatment options.

The reduction in hospitalisation and severe RSV illness among infants born to vaccinated mothers was the most consistent clinical finding and was observed across several vaccine formulations, pointing to the potential of maternal vaccination to reduce RSV-associated healthcare utilisation in early life. Crucially, real-world efficacy estimates from national and multicenter programs in Argentina and the US that were unavailable during the initial search now support this decrease (Moline et al., 2026; Pérez Marc et al., 2025).

No significant difference in infant mortality was observed between vaccine and placebo groups; this is consistent with the low baseline mortality rate in the included studies and their limited statistical power to detect differences in rare outcomes. Crucially, there was no indication of increased mortality, which is encouraging for the overall safety profile. However, this should be interpreted cautiously rather than as definitive assurance due to the large confidence interval surrounding the single reported mortality effect estimate (Dieussaert et al., 2024).

In line with more general antimicrobial stewardship objectives, the observed decrease in antimicrobial prescribing during early infancy (Lewnard et al., 2022) is a potentially significant indirect benefit of maternal vaccination; however, as previously mentioned, this finding is based on a single post-hoc analysis of a single vaccine formulation and requires confirmation before being generalised.

Preterm birth results should not be summed up as a single effect because they varied significantly by vaccination formulation and experiment. The clinical development effort for that vaccine candidate was discontinued after the GSK unadjuvanted RSVpreF study revealed a statistically significant increase in preterm birth (Dieussaert et al., 2024).

In contrast, a statistically significant rise in preterm birth was not observed in post-hoc and subset analyses of the unique Pfizer bivalent RSVpreF (MATISSE) vaccination (Li et al., 2022; Otsuki et al., 2024). This disparity emphasises that formulation-specific and trial-specific results should inform clinical and regulatory interpretation, and that preterm-birth risk cannot be taken to be a class impact of RSVpreF vaccinations in general.

Maternal safety data were otherwise reassuring. Local reactogenicity – erythema, pain, and edema at the injection site – was more frequent in vaccinated mothers, consistent with expected immune activation following vaccination, but these reactions were predominantly mild to moderate and self-limiting. Systemic symptoms such as vomiting and fatigue were reported more commonly in vaccinated mothers, while fever, headache, joint pain, and gastrointestinal symptoms including diarrhea were generally similar across groups. There was no increase in the rate of serious maternal adverse events.

These findings are broadly consistent with emerging evidence supporting maternal immunisation as a strategy for protecting infants against RSV, complementing other preventive approaches such as monoclonal antibody prophylaxis (e.g. nirsevimab). Through the transplacental transfer of passive antibody, maternal vaccination provides infants with immediate protection during the early period of life, when RSV morbidity is highest.

The Cochrane systematic review by Phijffer et al. (2024), which combined six RCTs (17,991 pregnant women) and found that maternal RSV vaccination reduces RSV-related infant hospitalisation with little to no effect on birth defects and probably little to no effect on fetal growth restriction, is directionally consistent with the efficacy findings.

The current review expands this trial-based evidence base with real-world effectiveness data from Argentina, the United Kingdom, Scotland, and the United States (McLachlan et al., 2025; Moline et al., 2026; Pérez Marc et al., 2025; Williams et al., 2025), which broadly corroborate the trial-based efficacy estimates in more diverse populations and healthcare settings. This is because Phijffer et al. limited eligibility to randomised trials.

The rapid meta-analysis of preterm birth by Kuitunen et al. (2025), which combined six RCTs and two observational studies, found higher odds of preterm birth in the randomised evidence (OR 1.17, 95% CI 1.02–1.34) but no such association in the smaller observational evidence available at the time (OR 0.93, 95% CI 0.69–1.25) for safety.

With the addition of two much larger real-world cohorts in this revision, Son et al. (2024) (2,973 pregnant individuals) and, in particular, Gabet et al. (2025), this review is able to significantly strengthen the observational side of this comparison and arrive at a similar overall picture: an isolated preterm-birth signal in one large RCT (Dieussaert et al., 2024) that has not been consistently replicated.

The largest maternal RSVpreF safety dataset to date, a countrywide French matched cohort of over 54,000 women, demonstrated no discernible increase in preterm birth among women vaccinated within the generally advised 32–36-week window. When combined with the trial-based post-hoc and subset studies that demonstrated the bivalent RSVpreF formulation did not significantly raise the risk of preterm birth (Li et al., 2022; Otsuki et al., 2024),

Although ongoing surveillance is still necessary, this consistent real-world evidence gives confidence that the isolated RCT-level signal seen with the unadjuvanted formulation is unlikely to generalise across RSVpreF vaccines when vaccination is administered in accordance with current recommendations.

Participants from a wide gestational range of 24–36 weeks were enrolled in the key MATISSE trial and a number of the other included RCTs (Kampmann et al., 2023; Madhi et al., 2020). Regulatory and advisory bodies in a number of jurisdictions subsequently limited the routine use of RSVpreF to 32–36 weeks’ gestation after reviewing the preterm-birth imbalance seen with vaccination earlier in this range. Several other national programs have since adopted this window, including Argentina (32 + 0/7–36 + 0/7 weeks, per national regulatory authorisation) and France (32–36 weeks, per the 2024–2025 national immunisation campaign).

The BERNI efficacy research in Argentina (Pérez Marc et al., 2025), the French national safety cohort (Gabet et al., 2025), and the US surveillance data (Moline et al., 2026) are examples of real-world studies that were mostly carried out inside this more limited, currently advised window.

Their reassuring safety findings – no significant excess of preterm birth, stillbirth, or small-for-gestational-age birth weight among women vaccinated within this window – together with sustained real-world effectiveness against infant RSV hospitalisation, support the specific policy of vaccination at 32–36 weeks rather than the broader gestational range originally studied in the earliest trials.

### 4.1. Implications for policy

These findings mostly align with the direction of international consensus and advisory guidelines endorsing maternal immunisation methods for early infant protection (WHO, 2024). Incorporating maternal RSV vaccination programmes, particularly in countries with the highest RSV burden such as LMICs, may meaningfully advance global child health objectives. Ongoing surveillance and post-implementation studies will be important to monitor long-term safety and effectiveness and to ensure equitable access to vaccination.

Although not a formal efficacy or safety outcome and thus not included in the data-extraction tables, the combined uptake of nirsevimab and maternal RSVpreF vaccination exceeded 85% in a single US military treatment facility with universal healthcare access (Homo et al., 2024). This finding is informative for program planning in comparable universal-access settings. High real-world uptake appears achievable even outside dedicated research settings.

### 4.2. Strengths and limitations

Large multicenter RCTs and real-world observational studies covering a wider range of nations are included in this study, which improves the findings’ generalizability. However, there are a number of limitations that should be taken into account: (1) the search was limited to English-language publications, which may have introduced language or publication bias; (2) several outcomes were reported in overlapping populations because multiple included publications originate from the same parent trials; although unique-trial totals take this into account, overlapping participants continue to be a potential source of double-weighting in qualitative synthesis; (3) The majority of included RCTs were sponsored by the industry (Pfizer, GSK, Novavax), which may introduce sponsorship-related bias; (4) statistical power was limited for rare maternal and neonatal adverse events, such as mortality; (5) follow-up beyond the first year of infant life was either limited or nonexistent in nearly all included studies; (6) significant heterogeneity existed in vaccine formulation, antigen dose, adjuvant use, and gestational-age window of administration; (7) an independent risk-of-bias assessment revealed a higher risk of selective outcome reporting bias in several post-hoclonal antibody prophylaxis over the course of a full RSV season.

## 5. Conclusions

Maternal RSV vaccination was linked to significant protection against hospitalisation in the early stages of infancy and severe RSV illness. Real-world data from national programs in Argentina, France, and the United States now supports the conclusion that there was no discernible increase in major maternal or neonatal adverse events when vaccination took place within the currently advised 32–36-week gestational window.

Continued, formulation-specific post-marketing surveillance is supported by variations in vaccine formulations and findings regarding preterm birth, particularly the isolated signal seen with the unadjuvanted GSK RSVpreF formulation, which has not been repeated in bivalent-formulation or real-world data.

Mothers who received vaccinations were more likely to experience mild, temporary local and systemic responses. When considered collectively, the data points to a positive benefit-risk profile for maternal RSV vaccination and emphasises the ongoing significance of formulation-specific safety monitoring.

## Data Availability

The datasets generated and/or analyzed during the current study are available from the corresponding author upon reasonable request.
